# Costs of inpatient hospitalisations in the last year of life in older New Zealanders: a cohort study

**DOI:** 10.1186/s12877-021-02458-6

**Published:** 2021-09-27

**Authors:** Oliver W. Scott, Merryn Gott, Richard Edlin, Simon A. Moyes, Marama Muru-Lanning, Ngaire Kerse

**Affiliations:** 1 School of Population Health, University of Auckland, 85 Park Road, Grafton, Auckland, 1023 New Zealand; 2 School of Nursing, University of Auckland, 85 Park Road, Grafton, Auckland, 1023 New Zealand; 3 James Henare Research Centre, University of Auckland, 18 Wynyard Street, Auckland Central, Auckland, 1010 New Zealand

**Keywords:** Older people, Hospitalisation costs, Last year of life, Health equity, Māori

## Abstract

**Background:**

Rapidly ageing populations means that many people now die in advanced age. This paper investigated public hospital and long-term care home costs in the 12 months before death in Māori and non-Māori of advanced age in New Zealand.

**Methods:**

Data from an existing longitudinal study (LiLACS NZ) was used, in which 937 older New Zealanders were enrolled in 2010. At the time of this study, 213 Māori and 241 non-Māori in the cohort had died. National Health Index numbers were linked to the hospitalisation National Minimum Dataset to ascertain public hospitalisation and care home costs in the last year of life.

**Results:**

The average total publicly funded hospital and long-term care home costs in the 12 months prior to death were $16,211 and $17,351 for Māori and non-Māori respectively. Non-Māori tended to have long lengths of stay in their last year of life, and non-Māori men had the highest proportion with high costs and long lengths of stay in care homes. Costs in the last year of life were 8.1 times higher in comparison to costs for individuals who did not die in the same time period.

**Conclusion:**

Despite New Zealand’s commitment to providing an equitable level of healthcare, this study illustrated that ethnic and gender disparities are still apparent at the end of life. This raises questions as to whether money at the end of life is being spent appropriately, and how it could potentially be more equitably targeted to meet the diverse needs of older people and their families.

**Supplementary Information:**

The online version contains supplementary material available at 10.1186/s12877-021-02458-6.

## Background

Rapidly ageing populations means that many people now die in advanced age, and the number of deaths in advanced age is projected to increase substantially over the coming decades in most countries [1]. Countries where health costs are predominantly publicly funded will experience greater costs to the tax base over the next decades, however the extent of these costs is not known.

Understanding the costs of health services in the last year of life is important for several reasons. Firstly, there is evidence that the inverse care law applies at the end of life, meaning that people most in need of care often miss out [2]. Crucially, this pattern of care is determined in no small part by social determinants such as cultural and ethnic affiliation, gender, and socioeconomic status [2]. Therefore, understanding cost patterns is an important factor in addressing established inequities in healthcare [3–6]. Secondly, end of life healthcare costs are dominated by costs incurred from inpatient hospitalisations [7, 8]. However, there is debate as to the extent to which this pattern of healthcare utilisation results in real benefit to the patient [9], and if it reflects the end of life aspirations of people of advanced age [10]. Finally, it is essential that governments are prepared for the level of investment required to support current patterns of end of life healthcare utilisation.

Although a high level of healthcare utilisation is common in the last year of life, this variation has not been sufficiently described in New Zealand. In light of population projections estimating a 88% increase in the number of older Māori (the indigenous people of NZ, with older people being defined as those aged 80+), and a 55% increase in the number of older non-Māori in the next 10 years in NZ, as well an increase in the number of deaths per year in NZ by 30% in the next 15 years [11, 12], this paper aims to establish public hospital (acute) and long-term aged residential care home costs in the 12 months before death in Māori and non-Māori of advanced age in NZ. It also aims to establish the primary correlates of high costs in the last year of life.

## Methods

### Population and data sources

This paper draws on data from an existing longitudinal study in New Zealand (LiLACS NZ), as well as data from matching participants National Health Index (NHI) numbers to the hospitalisation National Minimum Dataset (NMDS) held by the Ministry of Health (MoH). When LiLACS NZ was initiated in 2010, 937 older New Zealanders were enrolled from a population-based sample, including 421 Māori aged between 80 and 90, and 516 non-Māori all aged 85 [13]. In this cohort, the non-Māori group was overwhelmingly made up of Europeans, with 89% being NZ European, and 10% Other European [14]. All participants were followed up and NHI matching enabled notification of deaths. At the time of this study, 454 individuals in the cohort had died, 213 of whom were Māori and 241 non-Māori.

Covariates on study participants were gathered in the first wave of data collection, including gender, age, region, district health board (regional funding body) of residence (DHB, Bay of Plenty or Lakes), and living arrangement (alone, with spouse only, or other). Deprivation (10 deciles regrouped into 3 categories) was based on the NZDep, an area level index ascertained by geocoding participants addresses at the time of enrolment [15]. Health conditions (asthma, cancer, cerebrovascular accident, congestive heart failure, and diabetes) were ascertained from a combination of self-report, physical examination, hospital and primary care medical record review, and blood analyses [16].

The NMDS contains admission data on acute public hospital admissions and long-term aged residential care admissions (henceforth termed care homes). Care homes in New Zealand consist of both high-level dependency (similar to nursing home care in the United Kingdom and Australia and skilled nursing facility care in the United States of America) and low-level dependency (similar to residential homes in the UK, hostels in Australia, and assisted living care in the USA). Based on a standard financial assessment, high dependency care home costs are fully subsidised in NZ, and low dependency care home costs are part subsidised based on a means test. In this paper, we report MoH (publicly) funded costs as recorded in the NMDS. Care home costs reported are those relating to high-level dependency (i.e. hospital level aged residential care) only.

Acute public hospitalisation costs for each participant were calculated based on the Weighted Inlier Equivalent Separations (WIES) attached to each hospitalisation event. These WIES are calculated based on length of stay and diagnostic-related groups [17], with individual level data available on request from the MoH. Costs are assigned by multiplying the number of WIES by a standard cost weight specific to a year, in this case 2016. Yearly costs relating to admissions in care home facilities were based on the total average annual cost of continuing care in aged residential facilities in 2018 ($77,410) [18], adjusted to 2016 NZ dollars by removing the inflation observed in WIES costs between 2016 and 2018 (Additional file 1) [6, 17]. Individual care home costs were calculated by applying the annualised costs pro rata to the days in care from the MoH data.

Costs were reported in two categories, these being acute public hospital costs and long-term care home costs. As we are taking a health services costs approach to our study, we do not report other private costs such as those relating to primary care or out of pocket costs for care homes.

### Statistical analysis

Māori and non-Māori are reported separately to examine potential inequities and because care practices differ between cultural groups. Descriptive statistics illustrate costs in the last year of life stratified by sociodemographic and health variables (Table 1a and b). We tested for differences between different levels of variables within the same ethnic group. Table 2 highlights the distribution of costs among all participants stratified by ethnicity and gender. Similar analyses were carried out examining the distribution in the ‘number of admissions’ and ‘length of stay’ variables. We categorised each of these three variables into three groups each. Costs were categorised into no cost, moderate cost (between $1 and $50,000), and high cost ($50,000 and over). The number of admissions variable was categorised into no admissions, between 1 and 4 admissions, and 5 or more admissions, and the length of stay variable was categorised into no stay, between 1 and 39 days in hospital, and 40 or more days in hospital.

**Table 1:** Māori and non-Māori costs in the last year of life

A			
		Māori (n, SD)	non-Māori (n, SD)
Overall Cost		16,211 (213, 17,075)	17,351 (241, 20,520)
Gender	Male	16,475 (106, 14,928)	20,320 (126, 23,417) ^a^
	Female	15,948 (107, 19,032)	14,098 (115, 16,271) ^a^
DHB	BoP	16,457 (158, 17,933)	17,938 (210, 21,040)
	Lakes	15,503 (55, 14,452)	13,373 (31, 16,298)
Region	Rural	15,705 (67, 14,688)	17,021 (150, 18,655)
	Urban	16,263 (47, 15,523)	18,891 (24, 23,534)
NZDep group	1	12,451 (33, 13,030)	19,494 (61, 19,624)
	2	18,327 (54, 20,305)	18,696 (95, 24,243)
	3	16,288 (126, 16,447)	14,310 (85, 15,985)
Living arrangement	Alone	13,128 (53, 14,276)	14,731 (81, 15,905) ^a^
	With spouse only	19,040 (21, 13,633)	21,743 (65, 22,296) ^a^
	Other	17,918 (48, 15,968)	14,452 (30, 19,578) ^a^
Health conditions	Asthma	17,259 (86, 18,343)	15,409 (79, 20,223)
	No asthma	15,501 (127, 16,196)	18,298 (162, 20,659)
	Cancer	13,504 (46, 14,525)	20,305 (121, 21,763) ^a^
	No cancer	16,956 (167, 17,679)	14,372 (120, 18,809) ^a^
	Cerebrovascular accident (stroke)	18,306 (61, 22,100)	17,556 (81, 20,719)
	No CVA	15,370 (152, 14,587)	17,247 (160, 20,483)
	Congestive heart failure	20,564 (84, 18,835) ^a^	19,263 (72, 20,277)
	No CHF	13,376 (129, 15,241) ^a^	16,536 (169, 20,628)
	Diabetes	19,844 (75, 20,242) ^a^	19,764 (50, 24,170)
	No diabetes	14,236 (138, 14,788) ^a^	16,719 (191, 19,475)
B			
		Māori (n, SD)	non-Māori (n, SD)
Overall cost		15,592 (213, 15,961)	15,372 (241, 18,774)
Gender	Male	16,373 (106, 14,957)	17,081 (126, 20,933)
	Female	14,817 (107, 16,932)	13,500 (115, 15,962)
DHB	BoP	15,622 (158, 16,497)	15,940 (210, 19,468)
	Lakes	15,503 (55, 14,452)	11,525 (31, 12,716)
Region	Urban	15,632 (67, 14,681)	15,416 (150, 16,541)
	Rural	16,211 (47, 15,567)	18,849 (24, 23,511)
NZDep group	1	12,277 (33, 13,047)	19,260 (61, 19,576)
	2	16,086 (54, 16,454)	15,220 (95, 21,091)
	3	16,248 (126, 16,440)	12,752 (85, 14,750)
Living arrangement	Alone	13,036 (53, 14,249)	14,287 (81, 15,625) ^a^
	With spouse only	19,040 (21, 13,633)	19,777 (65, 20,352) ^a^
	Other	17,867 (48, 16,014)	11,850 (30, 15,046) ^a^
Health conditions	Asthma	16,266 (86, 17,319)	14,606 (79, 20,112)
	No asthma	15,135 (127, 15,026)	15,746 (162, 18,139)
	Cancer	13,437 (46, 14,571)	17,624 (121, 19,589) ^a^
	No cancer	16,185 (167, 16,314)	13,102 (120, 17,706) ^a^
	CVA	16,410 (61, 19,038)	14,748 (81, 18,051)
	No CVA	15,263 (152, 14,604)	15,688 (160, 19,177)
	CHF	20,008 (84, 17,405) ^a^	17,592 (72, 19,124)
	No CHF	12,716 (129, 14,296) ^a^	14,426 (169, 18,599)
	Diabetes	18,268 (75, 17,691)	19,322 (50, 24,112)
	No diabetes	14,137 (138, 14,802)	14,338 (191, 17,031)

Notes for Table 1A

Note 1) Includes both public hospital (acute) and care home costs to the Ministry of Health

Note 2) Mean cost per person is shown

Note 3) All costs were summed and adjusted to 2016 costs in New Zealand dollars

Note 4) ^a^denotes a statistically significant difference between groups at the 0.05 level of significance. We tested for differences between different levels of variables within the same ethnic group. For example, there was a statistically significant difference found between non-Māori males and females

Note 5) *CVA* cerebrovascular accident, *CHF* congestive heart failure

Note 6) NZDep was categorised as the following: 1–4, 5–7, and 8–10, where 1 represents the areas with the least deprived scores and 10 the areas with the most deprived scores

Notes for Table 1B

Note 1) Includes public hospital (acute) costs to the Ministry of Health

Note 2) Mean cost per person is shown

Note 3) All costs were summed and adjusted to 2016 costs in New Zealand dollars

Note 4) ^a^denotes a statistically significant difference between groups at the 0.05 level of significance. We tested for differences between different levels of variables within the same ethnic group

Note 5) *CVA* cerebrovascular accident, *CHF* congestive heart failure

Note 6) NZDep was categorised as the following: 1–4, 5–7, and 8–10, where 1 represents the areas with the least deprived scores and 10 the areas with the most deprived scores

**Table 2:** Distribution of the cost, number of admissions, and length of stay in the last year of life variables

Variable	Public hospital, MoH funded	Care home, MoH funded	MM (n, %)	MW (n, %)	NMM (n, %)	NMW (n, %)
Cost in the last year of life	No cost	No cost	17 (16.0)	22 (20.6)	17 (13.5)	23 (20.0)
		Moderate cost	0 (0)	0 (0)	2 (1.6)	2 (1.7)
		High cost	0 (0)	1 (0.9)	3 (2.4)	0 (0)
	Moderate cost	No cost	83 (78.3)	75 (70.1)	85 (67.5)	81 (70.4)
		Moderate cost	3 (2.8)	3 (2.8)	10 (7.9)	5 (4.3)
		High cost	0 (0)	0 (0)	1 (0.8)	0 (0)
	High cost	No cost	3 (2.8)	5 (4.7)	7 (5.6)	3 (2.6)
		Moderate cost	0 (0)	0 (0)	1 (0.8)	1 (0.9)
		High cost	0 (0)	1 (0.9)	0 (0)	0 (0)
Number of admissions in the last year of life	No admissions	No admissions	17 (16.0)	24 (22.4)	19 (15.1)	24 (20.9)
		1+ admissions	0 (0)	0 (0)	3 (2.4)	2 (1.7)
	Between 1 and 4 admissions	No admissions	66 (62.3)	68 (63.6)	76 (60.3)	68 (59.1)
		1+ admissions	3 (2.8)	4 (3.7)	8 (6.3)	4 (3.5)
	5 or more admissions	No admissions	20 (18.9)	11 (10.3)	17 (13.5)	15 (13.0)
		1+ admissions	0 (0)	0 (0)	3 (2.4)	2 (1.7)
Length of stay in the last year of life	No stay in hospital	No stay in hospital	17 (16.0)	22 (20.6)	17 (13.5)	23 (20.0)
		Between 1 and 39 days in hospital	0 (0)	0 (0)	2 (1.6)	0 (0)
		40 or more days in hospital	0 (0)	1 (0.9)	3 (2.4)	2 (1.7)
	1–39 days in hospital	No stay in hospital	75 (70.8)	72 (67.3)	79 (62.7)	71 (61.7)
		Between 1 and 39 days in hospital	3 (2.8)	3 (2.8)	5 (4.0)	3 (2.6)
		40 or more days in hospital	0 (0)	0 (0)	5 (4.0)	1 (0.9)
	40 or more days in hospital	No stay in hospital	11 (10.4)	8 (7.5)	13 (10.3)	13 (11.3)
		Between 1 and 39 days in hospital	0 (0)	0 (0)	1 (0.8)	1 (0.9)
		40 or more days in hospital	0 (0)	1 (0.9)	1 (0.8)	1 (0.9)
Combined costs, both public hospital and care homes						
Cost in the last year of life		No cost	17 (16.0)	22 (20.6)	17 (13.5)	23 (20.0)
		Moderate cost	86 (81.1)	78 (72.9)	97 (77.0)	88 (76.5)
		High cost	3 (2.8)	7 (6.5)	12 (9.5)	4 (3.5)

Note 1) Includes both public hospital (acute) and care home costs to the Ministry of Health

Note 2) Moderate cost was between $1 and $50,000, and high cost was $50,000 and over

Note 3) *MM* Māori men, *MW* Māori women, *NMM* non-Māori men, *NMW* non-Māori women

In order to compare costs in those who died in the last 12 months against those who did not die in the same time period, participants were matched on birthdate and costs were compared between those who died and those still living. For example, if a participant died between their 85th and 86th birthdays, their last year of life costs were compared with the mean of all individuals who were still alive between their 85th and 86th birthdays. Age groups were grouped into 3-year age bands, and analysis was only carried out in each band if both groups had 10 or more participants. As individuals who did not die often had costs spanning multiple years, weighted averages were computed for this group. Comparisons between those who died and those still alive are presented as a ratio. Similar analysis was carried out for the number of admissions and length of stay variables.

We used generalised linear regression models to explore variables statistically significantly associated with costs in the last year of life. Due to the skewed distribution of the cost data it was found that using a log link rather than identity link improved the model fit markedly, meaning that log (costs) was the dependent variable for regression analysis. Dummy variables were created with non-Māori males as the comparison group, and GLM regression models were built in stages; the 4 ethnic/gender combinations, the previous model plus all variables except health conditions, and the previous model plus health conditions. Analyses were conducted using SAS 9.4 for Windows (© 2016 SAS Institute Inc., Cary, NC, USA).

## Results

The average total publicly funded hospital and long-term care home costs in the 12 months prior to death were $16,211 and $17,351 for Māori and non-Māori respectively (Table 1a, difference not significant). These costs are predominantly attributable to acute hospital costs ($15,592 and $15,372 for Māori and non-Māori respectively, Table 1b).

Within ethnic group comparisons show costs were higher for non-Māori men than non-Māori women, and costs were lower for non-Māori who lived alone. Costs were higher for non-Māori who had cancer than non-Māori who did not, and higher for Māori patients with congestive heart failure (CHF) and diabetes than Māori without these conditions (all *p* < 0.05, Table 1a). When considering acute hospitalisations alone, the only differences that remained were the following; for non-Māori, those living alone had lower costs and those with cancer had higher costs. For Māori, those with CHF had higher costs (all *p* < 0.05, Table 1b).

The majority of participants had moderate public hospital costs (up to $50,000) without care home costs (324, 71%), and 79 (17%) had no acute hospital or care home costs. Only 33 (7%) had any long-term residential care home costs reported in the NMDS (Table 2).

Twelve (9.5%) non-Māori men had high costs in their last year of life (≥$50,000), whereas 7 (6.5%) Māori women had high costs (Table 2), and 23 (18.3%) non-Māori men spent 40 or more days in a public hospital or care home in their last year of life, compared to 18 (15.7%) non-Māori women (see Supplementary Table 1, Additional file 2). The small number of non-Māori men with high costs and long lengths of stay may have skewed the data, largely caused by the few non-Māori men residing in care homes for long periods in their last year of life. Nine non-Māori men spent 40 or more days in care homes in their last year of life compared to 4 non-Māori women (Table 2). When considering public hospital admissions alone, 8 (6.3%) non-Māori men and 6 (5.6%) Māori women had high costs in their last year of life (see Supplementary Table 2, Additional file 2).

Costs in the last year of life were much higher in comparison to costs for individuals who did not die in the same time period (Table 3). The difference was larger for non-Māori women in comparison to any other ethnic/gender combination. The ratio of difference tended to be smaller when considering acute hospitalisations alone (see Supplementary Table 3, Additional file 2). Similar results were observed for the number of admissions and length of stay variables, with the ratio of difference largest for non-Māori women and non-Māori men respectively (see Supplementary Tables 4 and 5, Additional file 2).

**Table 3:** Average costs in the last year of life vs costs for those still living

	Age groups						
	80–82	82–84	84–86	86–88	88–90	90–92	Total
All ethnic groups/genders							
Last year of life	18,447 (14)	16,268 (30)	21,980 (77)	14,890 (122)	17,395 (111)	15,618 (81)	16,816 (454)
Not last year of life	1940 (863)	2258 (833)	2293 (756)	2199 (585)	1520 (417)	1755 (305)	2073 (863)
Ratio	9.5	7.2	9.6	6.8	11.4	8.9	8.1
Māori men							
Last year of life	18,175 (10)	15,247 (20)	19,758 (30)	20,094 (12)	12,334 (17)	17,044 (8)	16,475 (106)
Not last year of life	2724 (152)	2913 (132)	2766 (102)	3236 (76)	2382 (39)	4098 (18)	2873 (152)
Ratio	6.7	5.2	7.1	6.2	5.2	4.2	5.7
Māori women							
Last year of life	19,127 (4)	18,310 (10)	18,322 (19)	13,988 (31)	21,739 (20)	9766 (13)	15,764 (107)
Not last year of life	2111 (213)	1597 (203)	1745 (184)	1742 (118)	3015 (61)	2429 (30)	1906 (213)
Ratio	9.1	11.5	10.5	8.0	7.2	4.0	8.3
Non-Māori men							
Last year of life	–	–	34,131 (17)	16,387 (39)	21,042 (42)	16,329 (28)	20,320 (126)
Not last year of life	–	–	3113 (216)	2847 (177)	1428 (135)	1160 (107)	2375 (233)
Ratio	–	–	11.0	5.8	14.7	14.1	8.6
Non-Māori women							
Last year of life	–	–	15,575 (11)	12,570 (40)	12,581 (32)	17,018 (32)	14,098 (115)
Not last year of life	–	–	1810 (254)	1562 (214)	967 (182)	1551 (150)	1608 (265)
Ratio	–	–	8.6	8.0	13.0	11.0	8.8

Note 1) Includes both public hospital (acute) and care home costs to the Ministry of Health

Note 2) Mean cost per person is shown

Note 3) All costs were summed and adjusted to 2016 costs in New Zealand dollars

Note 4) The numbers in brackets denote n

Note 5) 19 people who died aged over 92 years are omitted due to small numbers

Regression analyses examining influences on costs in the last year of life showed that there were no variables statistically significantly associated with log (costs) and no differences between the gender/ethnic groups in unadjusted or adjusted models (see Supplementary Table 6, Additional file 2). In terms of model calibration for the regression models, including demographic variables (DHB, rurality, deprivation, and living arrangement) or both demographic variables and health conditions improved the model fit. However, the Akaike Information Criteria indicated that this was possibly at the expense of overfitting (i.e. making the model unnecessarily complicated). As such, the adjusted models are not such improvements that they are preferable over our original (uncorrected) model.

## Discussion

The average last year of life healthcare expenditure of older New Zealanders in this study was $16,816 NZD per person, lower than a previous NZ study which examined deaths in the last year of life at all ages ($21,100 NZD) [8]. International comparisons vary, with an Australian study reporting an average last year of life cost of $13,513 AUD for inpatient hospitalisations in people aged 65 years and over [19], and a USA study reporting an average of approximately $23,619 USD for inpatient hospitalisations (including skilled nursing facility care) in Medicare beneficiaries aged 65 years and over [20]. In the USA study [20], 61% of the overall healthcare expenditure for patients in their last year of life was related to inpatient hospitalisations. Considering we report hospital costs exclusively, the total costs would be even greater.

While there was no overall disparity relating to ethnicity or gender in the adjusted regression models, there appears to be a small number of non-Māori who have very long lengths of stay in their last year of life. These results are largely in contrast to the international literature, where ethnic minorities have a higher degree of healthcare utilisation than whites in the last year of life [21–23]. Internationally, racial and ethnic minorities generally receive fewer medical interventions throughout life, but this often seems to be reversed at life’s end. In New Zealand, ethnic disparities in health care utilisation and outcomes throughout life are well documented [4, 5]. Indeed, we show small numbers of non-Māori have long lengths of stay at the end of life, although the difference is largely explained by the differing use of care homes. Furthermore, ongoing LiLACS analyses shows that care home use for Māori in this group is half that of non-Māori [24]. There is evidence to suggest that part of this difference may be attributable to New Zealand’s history of institutionalised racism and greater socioeconomic deprivation for Māori [25, 26], although cultural differences in end of life care, such as care for Māori at home and the use of traditional, non-Western medicines are likely to play a role as well [27–30]. Other LiLACS NZ reports have shown that costs to the public health system are ‘passed’ on to informal caregivers such as family and friends more so for Māori than non-Māori [27, 31], and as Hayman and others [32] note, while the costs of informal care are difficult to quantify, the value of such care can be substantial [33–35].

Participants who died cost 8.1 times more than those living on in the same year, which is slightly lower than a ratio of 8.7 found in the USA [20]. While a Danish study [36] found that costs differed (at all ages) between men (ratio of 13.3) and women (ratio of 9.4), we found a ratio of 8.6 for non-Māori men and 8.8 for non-Māori women. Although the ratio was lower for non-Māori men, this can be explained by higher costs at any stage of life relative to non-Māori women (i.e. NMM receive more health care resources in their last year of life as well as in years they do not die). Men are more likely than women to make contact with medical specialists and be admitted to hospital, and therefore receive more resources than women on average [37]. Moreover, non-Māori men were more likely than non-Māori women to express a preference for medical intervention at the end of life within this sample, consistent with evidence that men receive more chemotherapy and ICU admissions prior to death than women [38]. Medicare expenditure on inpatient services in the United States is also greater for men than women, which may be partly explained by women being more reliant on “social supportive” services than inpatient care [23]. In addition, earlier age at death is associated with greater spending, and men die younger than women on average [23].

We were unable to illustrate a strong trend toward older decedents costing less than their younger counterparts. Other literature has highlighted age disparities related to choice, care patterns, and care rationing [8, 23, 39–41]. As a result, some studies have noted that the deceased to surviving cost ratio often seems to diminish over time [36]. It is possible that we did not observe the same effect due to the advanced age group in our study.

Our regression model did not show any variables which were statistically significantly associated with costs. Although Hanchate and others [21] did not transform their cost variable into log (costs), their regression model included many variables which were statistically significant (e.g. race, health conditions, and deprivation), even when controlling for a number of similar covariates to our study. We suggest that the differences found in mean costs in Table 1 may be related to skew in the data, and indeed, the ‘last year cost’ and ‘length of stay’ variables were both highly skewed (partly shown in Table 2, see Supplementary Figures 1 and 2, Additional file 2), especially in non-Māori men. Chan and others [8] also found a small proportion of individuals with an unusually high level of healthcare utilisation in their last year of life to account for a significant proportion of the total cost, and it is worthy to note that these outliers are of importance in clinical practice [42].

This is the beginning of an exploration to ensure equitable and appropriate end of life care is delivered in NZ. Higher healthcare costs at the end of life may not necessarily translate to improvements in health outcomes, quality, access, or satisfaction with care [8, 43]. According to Chan and others [8], the use of high cost interventions should be based on clinical factors, patient’s expectations, and the cost utility of interventions. In this paper, we considered publicly funded costs exclusively and did not account for costs to patient’s families or other parts of the health system. As different groups may have different needs requiring different types of care and support at a system level, it is important that a holistic approach to patient and family healthcare is considered, while simultaneously respecting ethnic and gender specific care patterns. For example, LiLACS NZ participants highlighted that “not being a burden to family” is the most highly prioritised end of life preference for both Māori and non-Māori [44]. Internationally, some patient surveys suggest that around half of persons with a serious chronic illness would prefer to die at home rather than in hospital [45]. Other literature highlights more preventative strategies, such as the need to implement and evaluate interventions that are known to reduce hospitalisations [7, 46]. Given the different cultural approaches to end of life care, it is likely that a mixture of these strategies will be needed in the NZ context.

The primary strengths of our study are that we enrolled a relatively large number of people of advanced age using a population-based strategy, had an acceptable response rate at the studies outset, and were able to access most participant’s administrative health records. This allowed us to calculate hospitalisation costs in the last year of life and compare these with individuals still living. Furthermore, the focus on advanced age and equal explanatory power for Māori make this study novel and of adequate size for conclusions to be drawn. As this data is part of the wider LiLACS study, the results here can be cross-checked in future against other findings from the wider study and can contribute to a much richer set of results than possible from purely administrative data.

Conversely, the main limitation of our study is that we explored publicly funded healthcare costs exclusively and no other costs such as out of pocket care home costs, primary care costs, or informal care. We expect care home costs to be underestimated as we cannot be certain that all public funds for care home admissions are counted. As mentioned however, inpatient costs generally dominate expenditure at the end of life, so it is likely we captured the majority (or much more) of healthcare expenditure among our study population. Furthermore, it must be acknowledged that there is considerable variability in hospitalisation cost data and that results need to be interpreted with caution. While there was a suggestion that non-Māori men had higher costs and longer lengths of stay in their last year of life than other groups, it is possible that our results were driven by chance alone. The results from this study are suggestive of underlying differences between groups that warrant further investigation, such as using data from New Zealand’s Integrated Data Infrastructure. A further analysis of this administrative data would allow for a greater sample size and stronger regional lens as care access, utilisation, and underlying health structures may differ across the country.

This study set out to investigate costs in the last year of life in Māori and non-Māori of advanced age in NZ. Acute hospitalisation and care home costs were 8.1 times greater for patients in their last 12 months of life compared to those still living. There were ethnic and gender disparities highlighted. This raises questions as to whether money at the end of life is being spent appropriately, and how it could potentially be more equitably targeted to meet the diverse needs of older people and their families.

## Supplementary Information

**Additional file 1:** Calculation of the costs of hospital-level care in residential settings in New Zealand.

**Additional file 2: Supplementary Table 1.** Distribution of the cost, number of admissions, and length of stay in the last year of life variables. **Supplementary Table 2.** Distribution of the cost, number of admissions, and length of stay in the last year of life variables. **Supplementary Table 3.** Average costs in the last year of life vs costs for those still living. **Supplementary Table 4.** Average number of admissions in the last year of life vs admissions for those still living. **Supplementary Table 5.** Average length of stay in the last year of life vs length of stay for those still living. **Supplementary Table 6.** Generalised linear regression models predicting costs in the last year of life. **Supplementary Figure 1.** Distribution of the cost in the last year of life variable. **Supplementary Figure 2.** Distribution of the length of stay in the last year of life variable.

## Data Availability

The data regarding hospitalisations that support the findings of this study are available from the Ministry of Health (NZ), but restrictions apply to the availability of these data, which were used under license for the current study, and so are not publicly available. Data are however available from the authors upon reasonable request and with permission of the Ministry of Health (NZ). LILACS NZ data is available upon request for collaborations with NK and is subject to an approval process.

## Abbreviations

NHI: National Health Index numbers
NMDS: National Minimum Dataset
MoH: Ministry of Health
WIES: Weighted Inlier Equivalent Separations
