# Correction to: Costs of inpatient hospitalisations in the last year of life in older New Zealanders: a cohort study

**DOI:** 10.1186/s12877-021-02582-3

**Published:** 2021-12-15

**Authors:** Oliver W. Scott, Merryn Gott, Richard Edlin, Simon A. Moyes, Marama Muru-Lanning, Ngaire Kerse

**Affiliations:** 1 School of Population Health, University of Auckland, 85 Park Road, Grafton, Auckland, 1023 New Zealand; 2 School of Nursing, University of Auckland, 85 Park Road, Grafton, Auckland, 1023 New Zealand; 3 James Henare Research Centre, University of Auckland, 18 Wynyard Street, Auckland Central, Auckland, 1010 New Zealand

**Correction to BMC Geriatr 21, 514 (2021).**

**https://doi.org/10.1186/s12877-021-02458-6**

Several errors were introduced during publication of the original article [1]. These errors are as follows:

1. Additional file 1 (**Calculation of the costs of hospital-level care in residential settings in New Zealand**) and the in-text citation of this file were missing.
2. Additional file 1 (**Supplementary Tables and Figures**) should be Additional file 2.
3. The footnote clarifying NZDep groups was missing in Table 1 and Additional file 2:
  - NZDep was categorised as the following: 1–4, 5–7, and 8–10, where 1 represents the areas with the least deprived scores and 10 the areas with the most deprived scores.

These changes do not impact the conclusion of the article. The original article has been updated to rectify these errors. The publisher apologizes to the authors and readers for the inconvenience.
