# Correction to: Costs of inpatient hospitalisations in the last year of life in older New Zealanders: a cohort study

**DOI:** 10.1186/s12877-021-02735-4

**Published:** 2022-02-17

**Authors:** Oliver W. Scott, Merryn Gott, Richard Edlin, Simon A. Moyes, Marama Muru-Lanning, Ngaire Kerse

**Affiliations:** 1 School of Population Health, University of Auckland, 85 Park Road, Grafton, Auckland, 1023 New Zealand; 2 School of Nursing, University of Auckland, 85 Park Road, Grafton, Auckland, 1023 New Zealand; 3 James Henare Research Centre, University of Auckland, 18 Wynyard Street, Auckland Central, Auckland, 1010 New Zealand

**Correction to: BMC Geriatr 21, 514 (2021)**

**https://doi.org/10.1186/s12877-021-02458-6**

After publication of the article [1], the authors notified the publishers that the Supplementary Information in the HTML version of the article was incorrectly reported. These errors have now been rectified.

The original article [1] has been updated.
