# Supplementary material for: Costs of inpatient hospitalisations in the last year of life in older New Zealanders: a cohort study
Source: BMC Geriatr. 2021 Sep 27;21:514. doi: 10.1186/s12877-021-02458-6 (PMC8477539; doi:10.1186/s12877-021-02458-6)
Supplement: Supplementary file 1 — Additional file 1: Calculation of the costs of hospital-level care in residential settings in New Zealand. [file 12877_2021_2458_MOESM1_ESM.docx]

**Supplementary file 2. Calculation of the costs of hospital-level care in residential settings in New Zealand.**

In New Zealand, care is administered through the “Aged Residential Hospital Specialised Services Agreement” (ARHSS)^1^ between each provider and their respective DHBs, and the daily rates paid (after any contributions from residents) differ from facility to facility as a daily rate. The corresponding document “Age-Related Residential Care Services Agreement” (ARRC)^2^ deals with those who are in non-hospital level beds. There is no single figure that applies throughout the country and so it is only possible to obtain a figure by looking at nationwide figures, in whatever form these are reported. The 2019 Aged Residential Care Funding Model Review^3^ was conducted by EY and contains sufficient information to obtain a unit cost figure, albeit not one that has a strong adjustment for case-mix.

The ARRC figure applies to care before any ‘extra’ or ‘premium’ charges are levied, including those charged when providing care under the ARHSS. Page 78 of the EY report notes that the care category prices cover both the ARRC and ARHSS.

Figure 8 provides a 2018 median annual contract price. This suggests that continuing care receives an additional 58% per patient-year relative to rest homes, which equals $28,365. This suggests that a base payment to facilities is $28,365/0.58=$48,905.

In practice, there are three figures defined like this, and the estimate for aged residential care costs would be $17,656/0.36=$49,044 (dementia) and $38,857/0.79=$49,186 (psychogeriatric). Taking the average of these three figures, we suggest estimating the rest home payment as $49,045, and adding the previously stated additional $28,365 to this, the result is a cost per patient-year of $77,410.

This $77,410 corresponds (at 365.25 days) to around $211.94 per patient-day or $1,483.56 per patient-week in 2018 NZD.

A figure for 2016 was derived by correcting for the inflation observed in the price index for Weighted Inlier Equivalent Separations (WIES) between 2015/2016 and 2017/2018^4^ as 4921.16/4751.58=3.57%. Subtracting this inflation from the 2018 cost provides an estimated cost in 2016 NZD.

The 2016 cost for hospital-level residential care (after correction for inflation) was taken to be $204.63 per patient day.

**References**

^1^https://tas.health.nz/assets/Health-of-Older-People/ARHSS-Agreement-2021-2022-effective-1-August-2021-.pdf

^2^https://tas.health.nz/assets/Health-of-Older-People/ARRC-Agreement-2021-22-effective-1-August-2021-.pdf

^3^https://tas.health.nz/assets/Health-of-Older-People/ARC-Funding-Model-Review-Final-Report.pdf

^4^https://www.health.govt.nz/nz-health-statistics/data-references/weighted-inlier-equivalent-separations/wiesnz21-cost-weights
