# Supplementary material for: Costs of inpatient hospitalisations in the last year of life in older New Zealanders: a cohort study
Source: BMC Geriatr. 2021 Sep 27;21:514. doi: 10.1186/s12877-021-02458-6 (PMC8477539; doi:10.1186/s12877-021-02458-6)

**Supplementary Table 1. Distribution of the cost, number of admissions, and length of stay in the last year of life variables.**

| **Variable** | **Public hospital or care home, MoH funded** | **MM (n, %)** | **MW (n, %)** | **NMM (n, %)** | **NMW (n, %)** |
| --- | --- | --- | --- | --- | --- |
| Cost in the last year of life | No cost | 17 (16.0) | 22 (20.6) | 17 (13.5) | 23 (20.0) |
|  | Moderate cost | 86 (81.1) | 78 (72.9) | 97 (77.0) | 88 (76.5) |
|  | High cost | 3 (2.8) | 7 (6.5) | 12 (9.5) | 4 (3.5) |
| Number of admissions in the last year of life | No admissions | 17 (16.0) | 24 (22.4) | 19 (15.1) | 24 (20.9) |
|  | Between 1 and 4 admissions | 69 (65.1) | 70 (65.4) | 85 (67.5) | 73 (63.5) |
|  | 5 or more admissions | 20 (18.9) | 13 (12.1) | 22 (17.5) | 18 (15.7) |
| Length of stay in the last year of life | No stay in hospital | 17 (16.0) | 22 (20.6) | 17 (13.5) | 23 (20.0) |
|  | Between 1 and 39 days in hospital | 77 (72.6) | 73 (68.2) | 86 (68.3) | 74 (64.3) |
|  | 40 or more days in hospital | 12 (11.3) | 12 (11.2) | 23 (18.3) | 18 (15.7) |

*Note 1) Includes both public hospital (acute) and care home costs to the Ministry of Health.*

*Note 2) Moderate cost was between $1 and $50,000, and high cost was $50,000 and over.*

*Note 3) MM=Māori men, MW=Māori women, NMM=non-Māori men, NMW=non-Māori women.*

**Supplementary Table 2. Distribution of the cost, number of admissions, and length of stay in the last year of life variables.**

| **Variable** | **Public hospital, MoH funded** | **MM (n, %)** | **MW (n, %)** | **NMM (n, %)** | **NMW (n, %)** |
| --- | --- | --- | --- | --- | --- |
| Cost in the last year of life | No cost | 17 (16.0) | 23 (21.5) | 22 (17.5) | 25 (21.7) |
|  | Moderate cost | 86 (81.1) | 78 (72.9) | 96 (76.2) | 86 (74.8) |
|  | High cost | 3 (2.8) | 6 (5.6) | 8 (6.3) | 4 (3.5) |
| Number of admissions in the last year of life | No admissions | 17 (16.0) | 24 (22.4) | 22 (17.5) | 26 (22.6) |
|  | Between 1 and 4 admissions | 69 (65.1) | 72 (67.3) | 84 (66.7) | 72 (62.6) |
|  | 5 or more admissions | 20 (18.9) | 11 (10.3) | 20 (15.9) | 17 (14.8) |
| Length of stay in the last year of life | No stay in hospital | 17 (16.0) | 23 (21.5) | 22 (17.5) | 25 (21.7) |
|  | Between 1 and 39 days in hospital | 78 (73.6) | 75 (70.1) | 89 (70.6) | 75 (65.2) |
|  | 40 or more days in hospital | 11 (10.4) | 9 (8.4) | 15 (11.9) | 15 (13.0) |

*Note 1) Includes public hospital (acute) costs to the Ministry of Health.*

*Note 2) Moderate cost was between $1 and $50,000, and high cost was $50,000 and over.*

*Note 3) MM=Māori men, MW=Māori women, NMM=non-Māori men, NMW=non-Māori women.*

**Supplementary Table 3. Average costs in the last year of life vs costs for those still living.**

|  | Age groups | | | | | | |
| --- | --- | --- | --- | --- | --- | --- | --- |
|  | 80-82 | 82-84 | 84-86 | 86-88 | 88-90 | 90-92 | Total |
| **All ethnic groups/genders** |  |  |  |  |  |  |  |
| Last year of life | 18,447 (14) | 16,193 (30) | 19,038 (77) | 12,980 (122) | 16,200 (111) | 15,441 (81) | 15,432 (454) |
| Not last year of life | 1,922 (863) | 2,177 (833) | 2,186 (756) | 2,090 (585) | 1,500 (417) | 1,721 (305) | 2,007 (863) |
| Ratio | 9.6 | 7.4 | 8.7 | 6.2 | 10.8 | 9.0 | 7.7 |
| **Māori men** |  |  |  |  |  |  |  |
| Last year of life | 18,175 (10) | 15,135 (20) | 19,656 (30) | 20,094 (12) | 12,334 (17) | 16,353 (8) | 16,373 (106) |
| Not last year of life | 2,721 (152) | 2,913 (132) | 2,738 (102) | 3,216 (76) | 2,261 (39) | 4,098 (18) | 2,856 (152) |
| Ratio | 6.7 | 5.2 | 7.2 | 6.2 | 5.5 | 4.0 | 5.7 |
| **Māori women** |  |  |  |  |  |  |  |
| Last year of life | 19,127 (4) | 18,310 (10) | 14,247 (19) | 13,895 (31) | 19,703 (20) | 9,766 (13) | 14,633 (107) |
| Not last year of life | 2,094 (213) | 1,385 (203) | 1,742 (184) | 1,718 (118) | 3,015 (61) | 2,362 (30) | 1,840 (213) |
| Ratio | 9.1 | 13.2 | 8.2 | 8.1 | 6.5 | 4.1 | 8.0 |
| **Non-Māori men** |  |  |  |  |  |  |  |
| Last year of life | - | - | 26,747 (17) | 11,331 (39) | 19,009 (42) | 16,329 (28) | 17,081 (126) |
| Not last year of life | - | - | 2,899 (216) | 2,582 (177) | 1,418 (135) | 1,125 (107) | 2,264 (233) |
| Ratio | - | - | 9.2 | 4.4 | 13.4 | 14.5 | 7.5 |
| **Non-Māori women** |  |  |  |  |  |  |  |
| Last year of life | - | - | 13,715 (11) | 11,746 (40) | 12,376 (32) | 16,743 (32) | 13,500 (115) |
| Not last year of life | - | - | 1,687 (254) | 1,510 (214) | 954 (182) | 1,522 (150) | 1,561 (265) |
| Ratio | - | - | 8.1 | 7.8 | 13.0 | 11.0 | 8.6 |

*Note 1) Includes public hospital (acute) costs to the Ministry of Health.*

*Note 2) Mean cost per person is shown.*

*Note 3) All costs were summed and adjusted to 2016 costs in New Zealand dollars.*

*Note 4) The numbers in brackets denote n.*

*Note 5) 19 people who died aged over 92 years are omitted due to small numbers.*

**Supplementary Table 4. Average number of admissions in the last year of life vs admissions for those still living**.

|  | Age groups | | | | | | |
| --- | --- | --- | --- | --- | --- | --- | --- |
|  | 80-82 | 82-84 | 84-86 | 86-88 | 88-90 | 90-92 | Total |
| **All ethnic groups/genders** |  |  |  |  |  |  |  |
| Last year of life | 2.9 (14) | 3.2 (30) | 2.9 (77) | 2.4 (122) | 2.6 (111) | 2.4 (81) | 2.6 (454) |
| Not last year of life | 0.3 (863) | 0.4 (833) | 0.4 (756) | 0.4 (585) | 0.3 (417) | 0.3 (305) | 0.4 (863) |
| Ratio | 8.5 | 8.0 | 6.8 | 5.7 | 8.8 | 7.6 | 6.8 |
| **Māori men** |  |  |  |  |  |  |  |
| Last year of life | 3.0 (10) | 3.1 (20) | 2.8 (30) | 3.7 (12) | 2.3 (17) | 2.9 (8) | 2.8 (106) |
| Not last year of life | 0.5 (152) | 0.6 (132) | 0.5 (102) | 0.6 (76) | 0.5 (39) | 0.7 (18) | 0.6 (152) |
| Ratio | 5.8 | 5.2 | 5.8 | 6.1 | 4.4 | 4.1 | 5.1 |
| **Māori women** |  |  |  |  |  |  |  |
| Last year of life | 2.5 (4) | 3.4 (10) | 2.6 (19) | 2.3 (31) | 2.5 (20) | 1.6 (13) | 2.3 (107) |
| Not last year of life | 0.4 (213) | 0.3 (203) | 0.3 (184) | 0.4 (118) | 0.3 (61) | 0.6 (30) | 0.3 (213) |
| Ratio | 7.1 | 11.5 | 8.3 | 5.8 | 7.4 | 2.7 | 6.9 |
| **Non-Māori men** |  |  |  |  |  |  |  |
| Last year of life | - | - | 3.7 (17) | 2.0 (39) | 3.3 (42) | 2.7 (28) | 2.8 (126) |
| Not last year of life | - | - | 0.6 (216) | 0.6 (177) | 0.4 (135) | 0.3 (107) | 0.5 (233) |
| Ratio | - | - | 6.0 | 3.6 | 8.8 | 10.8 | 6.3 |
| **Non-Māori women** |  |  |  |  |  |  |  |
| Last year of life | - | - | 2.5 (11) | 2.5 (40) | 2.0 (32) | 2.3 (32) | 2.3 (115) |
| Not last year of life | - | - | 0.3 (254) | 0.3 (214) | 0.2 (182) | 0.2 (150) | 0.3 (265) |
| Ratio | - | - | 7.7 | 9.3 | 10.7 | 11.3 | 8.5 |

*Note 1) Includes both public hospital (acute) and care home admissions.*

*Note 2) Mean number of admissions per person is shown.*

*Note 3) The numbers in brackets denote n.*

*Note 4) 19 people who died aged over 92 years are omitted due to small numbers.*

*Note 5) Some of the ratios cannot be directly derived from the mean number of admissions per person due to rounding.*

**Supplementary Table 5. Average length of stay in the last year of life vs length of stay for those still living.**

|  | Age groups | | | | | | |
| --- | --- | --- | --- | --- | --- | --- | --- |
|  | 80-82 | 82-84 | 84-86 | 86-88 | 88-90 | 90-92 | Total |
| **All ethnic groups/genders** |  |  |  |  |  |  |  |
| Last year of life | 17.1 (14) | 15.9 (30) | 33.8 (77) | 22.3 (122) | 21.3 (111) | 17.1 (81) | 21.9 (454) |
| Not last year of life | 1.6 (863) | 2.2 (833) | 2.4 (756) | 2.1 (585) | 1.5 (417) | 1.9 (305) | 2.0 (863) |
| Ratio | 10.9 | 7.3 | 14.4 | 10.5 | 13.9 | 9.1 | 11.1 |
| **Māori men** |  |  |  |  |  |  |  |
| Last year of life | 12.4 (10) | 14.2 (20) | 18.1 (30) | 19.8 (12) | 11.6 (17) | 20.8 (8) | 15.6 (106) |
| Not last year of life | 1.9 (152) | 2.2 (132) | 1.7 (102) | 2.3 (76) | 2.3 (39) | 4.6 (18) | 2.1 (152) |
| Ratio | 6.4 | 6.6 | 10.5 | 8.5 | 5.2 | 4.5 | 7.4 |
| **Māori women** |  |  |  |  |  |  |  |
| Last year of life | 28.8 (4) | 19.5 (10) | 34.8 (19) | 14.6 (31) | 29.3 (20) | 10.0 (13) | 20.5 (107) |
| Not last year of life | 1.9 (213) | 2.3 (203) | 1.7 (184) | 1.6 (118) | 2.9 (61) | 2.2 (30) | 2.0 (213) |
| Ratio | 14.8 | 8.6 | 20.1 | 9.1 | 10.2 | 4.6 | 10.3 |
| **Non-Māori men** |  |  |  |  |  |  |  |
| Last year of life | - | - | 65.7 (17) | 37.3 (39) | 26.6 (42) | 17.2 (28) | 33.1 (126) |
| Not last year of life | - | - | 3.2 (216) | 3.0 (177) | 1.5 (135) | 1.2 (107) | 2.2 (233) |
| Ratio | - | - | 20.4 | 12.4 | 17.7 | 14.8 | 14.8 |
| **Non-Māori women** |  |  |  |  |  |  |  |
| Last year of life | - | - | 25.5 (11) | 14.5 (40) | 14.4 (32) | 19.0 (32) | 16.8 (115) |
| Not last year of life | - | - | 2.3 (254) | 1.6 (214) | 1.0 (182) | 1.8 (150) | 1.7 (265) |
| Ratio | - | - | 11.0 | 9.0 | 14.4 | 10.4 | 9.9 |

*Note 1) Includes both public hospital (acute) and care home admissions.*

*Note 2) Mean length of stay per person is shown.*

*Note 3) The numbers in brackets denote n.*

*Note 4) 19 people who died aged over 92 years are omitted due to small numbers.*

*Note 5) Some of the ratios cannot be directly derived from the mean length of stay per person due to rounding.*

**Supplementary Table 6. Generalised linear regression models predicting costs in the last year of life.**

| Variable | Model A, ethnic/gender combinations only | Pr > |t| | Pr > F | Model B (A + DHB, region, deprivation, and living arrangement) | Pr > |t| | Pr > F | Model C (B + health conditions) | Pr > |t| | Pr > F |
| --- | --- | --- | --- | --- | --- | --- | --- | --- | --- |
| Reference, constant | 9.92 | <0.0001 |  | 9.62 | <0.0001 |  | 9.31 | <0.0001 |  |
| **Ethnic/gender combination** |  |  |  |  |  |  |  |  |  |
| NMM | Reference |  | 0.51 | Reference |  | 0.97 | Reference |  | 0.95 |
| NMF | -0.37 | 0.14 |  | -0.11 | 0.68 |  | -0.16 | 0.57 |  |
| MM | -0.21 | 0.41 |  | 0.0012 | 0.997 |  | -0.030 | 0.94 |  |
| MF | -0.24 | 0.34 |  | -0.086 | 0.79 |  | -0.082 | 0.81 |  |
| **DHB** |  |  |  |  |  | 0.56 |  |  | 0.40 |
| Bay of Plenty |  |  |  | 0.17 | 0.56 |  | 0.25 | 0.40 |  |
| Lakes |  |  |  | Reference |  |  | Reference |  |  |
| **Region** |  |  |  |  |  | 0.91 |  |  | 0.82 |
| Urban |  |  |  | 0.18 | 0.18 |  | 0.064 | 0.82 |  |
| Rural |  |  |  | Reference |  |  | Reference |  |  |
| **NZDep group** |  |  |  |  |  | 0.85 |  |  | 0.68 |
| 1 |  |  |  | 0.14 | 0.64 |  | 0.16 | 0.61 |  |
| 2 |  |  |  | -0.022 | 0.93 |  | -0.12 | 0.65 |  |
| 3 |  |  |  | Reference |  |  | Reference |  |  |
| **Living arrangement** |  |  |  |  |  | 0.36 |  |  | 0.50 |
| Alone |  |  |  | -0.16 | 0.58 |  | -0.11 | 0.70 |  |
| With spouse only |  |  |  | 0.22 | 0.49 |  | 0.20 | 0.52 |  |
| Other |  |  |  | Reference |  |  | Reference |  |  |
| **Health conditions** |  |  |  |  |  |  |  |  |  |
| Asthma |  |  |  |  |  |  | -0.18 | 0.45 | 0.45 |
| Cancer |  |  |  |  |  |  | 0.15 | 0.52 | 0.52 |
| CVA |  |  |  |  |  |  | 0.046 | 0.86 | 0.86 |
| CHF |  |  |  |  |  |  | 0.36 | 0.14 | 0.14 |
| Diabetes |  |  |  |  |  |  | 0.23 | 0.39 | 0.39 |

*Note 1) Includes both public hospital (acute) and care home costs to the Ministry of Health.*

*Note 2) Log (costs in the last year of life) was the dependent variable.*

*Note 3) MM=Māori men, MW=Māori women, NMM=non-Māori men, NMW=non-Māori women.
Note 4) NZDep was categorised as the following: 1-4, 5-7, and 8-10, where 1 represents the areas with the least deprived scores and 10 the areas with the most deprived scores.*

**Supplementary Figure 1. Distribution of the cost in the last year of life variable.**


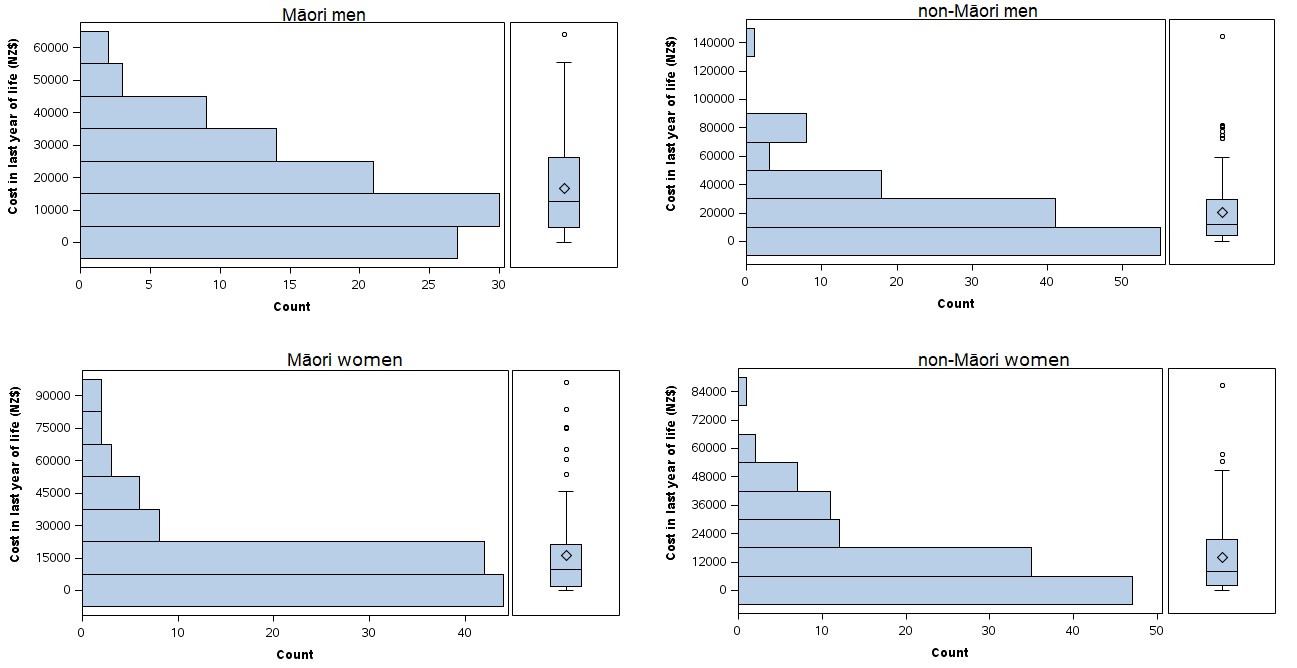


**Supplementary Figure 2. Distribution of the length of stay in the last year of life variable.**


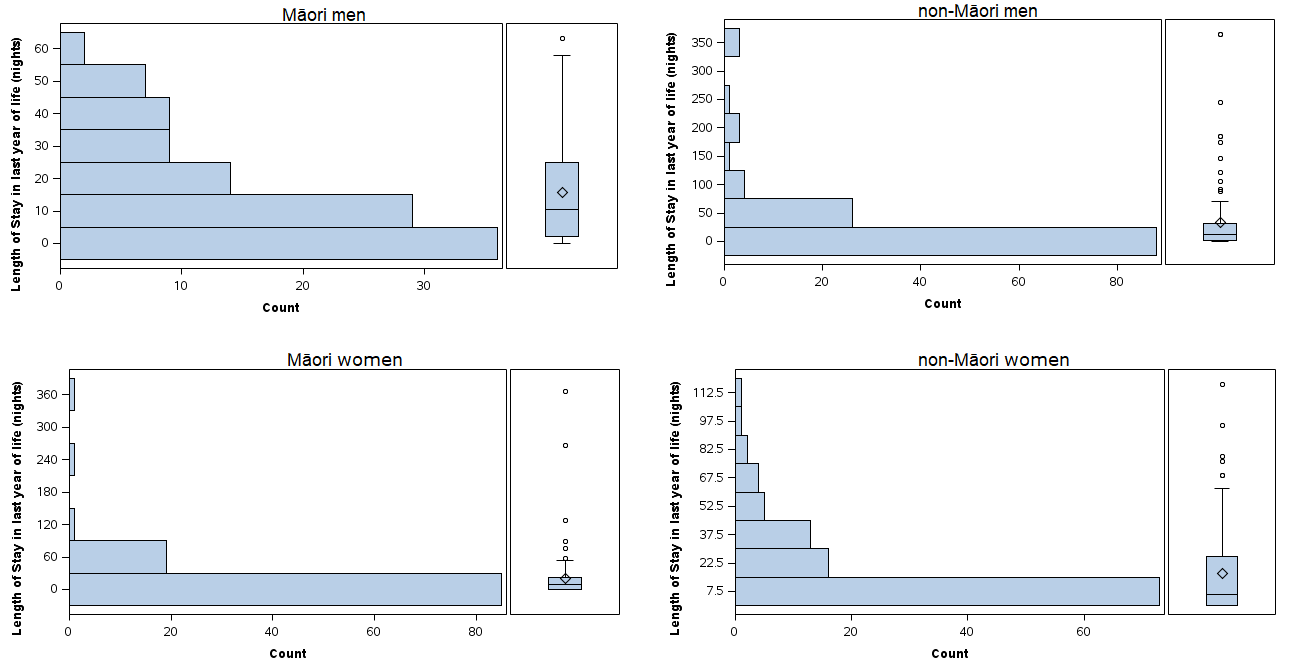

Supplement: Supplementary file 2 — Additional file 2: Supplementary Table 1. Distribution of the cost, number of admissions, and length of stay in the last year of life variables. Supplementary Table 2. Distribution of the cost, number of admissions, and length of stay in the last year of life variables. Supplementary Table 3. Average costs in the last year of life vs costs for those still living. Supplementary Table 4. Average number of admissions in the last year of life vs admissions for those still living. Supplementary Table 5. Average length of stay in the last year of life vs length of stay for those still living. Supplementary Table 6. Generalised linear regression models predicting costs in the last year of life. Supplementary Figure 1. Distribution of the cost in the last year of life variable. Supplementary Figure 2. Distribution of the length of stay in the last year of life variable. [file 12877_2021_2458_MOESM2_ESM.doc]
